# Submarine medicine as an analog for spaceflight: a review of acute medical care

**DOI:** 10.1038/s41526-026-00566-4

**Published:** 2026-01-30

**Authors:** Adam P. Prucka, Mark Shelhamer, Radames J. B. Cordero

**Affiliations:** 1 Department of Health Policy and Management, Johns Hopkins Bloomberg School of Public Health, Baltimore, MD 21205 USA; 2 Columbia Law School, Columbia University in the City of New York, New York, NY 10027 USA; 3 Department of Otolaryngology – Head and Neck Surgery, Johns Hopkins University School of Medicine, Baltimore, MD 21205 USA; 4 Department of Molecular Microbiology and Immunology, Johns Hopkins Bloomberg School of Public Health, Baltimore, MD 21205 USA

**Keywords:** Microbiology, Physiology, Diseases, Health care, Medical research

## Abstract

Long-duration spaceflight presents similar medical challenges to submarine deployment, including limited surgical capabilities and the psychological strain from isolation. This review synthesizes submarine medical literature to highlight parallels and divergences with space medicine, focusing on acute conditions. By comparing incidence data on acute medical diagnoses and examining case studies of these conditions, we propose countermeasures such as expanded surgical training and increased mental engagement. These analogies provide a blueprint for autonomous medical care on missions to the Moon and Mars.

## Introduction

Since the conclusion of the Apollo missions in 1972, human spaceflight has remained confined to Earth’s orbit, ranging from short-term Space Shuttle flights to longer stays aboard the International Space Station. However, space travel is soon poised to extend further from our planet, with NASA astronauts aiming to reach the Moon in this decade^1^ and to set their sights on Mars during the 2030 s.^2,3^ While these long-distance missions will represent significant technical accomplishments, they will also pose challenges for acute medical care on at least three levels. First, as space travel grows in length, the cumulative likelihood that an astronaut will experience an acute medical episode during the course of the mission will simultaneously increase.^4^ Second, the crew must be prepared to handle medical incidents with a high degree of autonomy, since the significant distance from Earth will challenge their ability to evacuate ailing astronauts in a timely fashion.^4^ Third, long-duration missions may threaten several of the determinants of health by disrupting ordinary sleep cycles,^5–7^ hindering adequate nutrition,^8^ and inhibiting mental health through prolonged isolation.^9^ Addressing these challenges will be essential to enable astronauts’ return to other worlds during this century.

To address these issues, researchers have drawn analogies to medical treatment in other extreme environments, from the bitter cold of the polar regions^10^ to the crushing depths of the oceans.^11^ One promising analog is military submarines, which, like spacecraft, house voyagers within an enclosed, self-contained capsule.^4^ For this reason, submarines provide a close parallel to many of the potential medical issues in long-duration space missions: the isolated underwater voyages can threaten sailors’ mental health,^12,13^ the need for secrecy on covert missions can hinder the feasibility of medical evacuations,^14^ and the abnormal lifestyle can impair the health of the crew.^15,16^ Hence, submarine medicine holds significant potential to inform medical care in outer space, helping space agencies to bolster their medical capabilities without reinventing existing approaches.

Admittedly, submarines have several key limitations as analogs to spacecraft. For instance, astronauts are generally more educated, older, and often have military officer experience,^17^ while submariners are typically younger, with 90% of the crew having an enlisted rank.^18^ Indeed, since empirical data shows that submarine officers experience substantially fewer medical incidents than enlisted sailors (36.3 vs. 62.5 for every 100 person-years), submarine medical data unstratified by military rank may overestimate the risk of medical incidents in space compared to studies that solely examine submarine officers.^17^ The distance to the surface of Earth is also greater for space travel, leading to longer communications delays and significantly greater challenges for medical evacuations.^19^ Additionally, astronauts must cope with the presence of cosmic radiation^20^ and the existence of microgravity, which may impact immune function^21,22^ and sleep^23^ and heighten the risk of injuries.^24,25^ Furthermore, while the lengthiest military submarine mission to date (204 days)^26^ is longer than an orbital Space Shuttle or lunar mission, it is still significantly shorter than a trip to Mars, which could approach 1000 days.^27^

Nevertheless, despite these limitations, no analog is perfect, and the significant parallels between submarines and spacecraft can still provide valuable strategies for strengthening acute medical care in enclosed, autonomous environments. Hence, this review seeks to outline the existing literature on submarine medicine and to demonstrate how its findings can inform the treatment of acute medical episodes, the provision of autonomous medical care, and the maintenance of a healthy lifestyle in long-duration space missions. Our findings, and our synthesis more broadly, emphasize acute medical events, reflecting the predominant focus of the primary data. Since the submarine medical literature is relatively lean and often decades-old, this review also serves as a call for renewed research in this field by demonstrating its importance to extreme environments beyond the underwater setting. By comprehensively uniting the depths and the heavens, this review showcases the value of the underwater frontier in guiding medical care in contexts beyond the surface of Earth.

## Acute medical conditions

Submariners and astronauts suffer from a wide range of acute health problems, some of which are prompted by the abnormal lifestyle and others of which are simply inherent to human existence. This section of the review examines these issues through a quantitative lens by presenting a comparison table (Table 1) which summarizes several studies that have provided epidemiological incidence data for diagnosed medical conditions aboard submarines. After describing this data, this section concludes with a discussion of minor medical problems that may not be reflected in diagnostic records, including self-medicated illnesses and dermatologic, vision, and dental conditions.

**Table 1:** Most common medical conditions aboard submarines

Rank	Tansey (% of all medical conditions)	Wilken (% of all medical conditions)	Thomas (% of all diagnoses)	Jan (% of all diagnoses)	Horn (% of all diagnoses)
1	General Surgery (21.13%)	General Medical (45.67%)	Respiratory (22.61%)	Injury/Poisoning (24.82%)	Injury (25%)
2	Bone/Joint (16.22%)	Orthopedic (12.41%)	Injury (15.65%)	Respiratory (18.34%)	Skin/Subcutaneous (16%)
3	General Medical (11.92%)	Ear, Nose, and Throat (12.19%)	Infections/Parasites (9.57%)	“Symptoms, Signs, & Ill-Defined Conditions” (15.47%)	Digestive (12%)
4	Gastrointestinal (11.06%)	Surgical (11.39%)	Musculoskeletal (8.70%)	Skin/Subcutaneous (8.63%)	Nervous (8% - tie)
5	Respiratory (7.86%)	Urological (6.38%)	Nervous/Sense Organ (7.83% - tie)	Infections/Parasites (9.57%)	Infections (8% - tie)
6	Urinary Tract (7.37%)	Dermatologic (3.87%)	Other (7.83% - tie)	Digestive (5.04%)	Respiratory (8% - tie)
7	Ear, Nose, and Throat (7.12%)	Eye (2.51%)	Digestive (6.96%)	Musculoskeletal/Connective (4.32% - tie)	Other (23%)*
8	Psychiatric (6.02%)	Dental (2.39%)	Circulatory (6.09% - tie)	Infections/Parasites (4.32% - tie)	-
9	Neurologic (4.18%)	Neurological and Neurosurgical (2.16%)	“Signs, Symptoms, & Ill-Defined Conditions” (6.09% - tie)	Genitourinary (3.60%)	-
10	Dental (3.56%)	Psychiatric (1.02%)	Skin/Subcutaneous (5.22%)	Mental Disorders (2.88%)	-
11	Eye (3.07%)	-	Genitourinary (3.48%)	“Supplementary Classification of Factors Influencing Health Status and Contact with Health Services” (2.16%)	-
12	Cardiovascular (0.49%)	-	“Non-Psychotic Mental Disorders” (0%)	“Endocrine, Nutritional, and Metabolic Diseases, and Immunity Disorders” (1.08%)	-
13	-	-	-	Circulatory (0.72% - tie)	-
14	-	-	-	Neoplasm (0.72% - tie)	-

Table 1 provides a summary of the medical conditions among military submariners, as reported by five epidemiological studies in the underwater realm. The medical conditions are represented in percentage format and ranked by their relative prevalence in each study population. While differences in the disease classification systems used in each study challenge direct comparisons, general trends can still be observed, especially the high prevalence of injuries, orthopedic disorders, and respiratory conditions. *Includes uncategorized diagnoses; composition not specified in original study.

Data Sources: Tansey^15^ (Appendices A1 through A12, analyzing the total number of cases in each diagnostic category from 1968-1973); Wilken^29^ (Table I and Tables III through XI, analyzing only “cases involving sick days” or “requiring admission to sick list”); Thomas^17^ (Table IV, analyzing officers age 30+ years); Jan^28^ (Table 1, analyzing the total of both primary and secondary diagnoses); Horn^18^ (p. 860).

### Relative incidence of medical problems

Tansey, Wilken, Thomas, Jan, and Horn provide detailed breakdowns of the medical conditions observed aboard submarines.^15,17,18,28,29^ We have represented their results side-by-side in Table 1, ranked by the relative percentages we have calculated for each diagnostic category. While Tansey provided data for both the 1963–1967 and 1968–1973 periods, we have only presented data from the latter period, since it accounts for the introduction of modern air filtration systems.^15^ Furthermore, since Horn’s diagnostic categories did not sum to 100%, we added an “other” row to fill this gap; however, the composition of this row is unknown.^18^ For Jan’s study, we analyzed the total of both primary and secondary diagnoses,^28^, and, for Thomas’ study, which stratifies by both age and rank, we only used the data among officers 30 years or older, since, although it is not a perfect demographic match, it likely provides the closest approximation of astronaut profiles.^17^

While the many differences in reporting conventions across these studies limit direct quantitative comparisons, their qualitative patterns offer valuable insights for extrapolating submarine health risks to spaceflight. Perhaps the most striking observation from Table 1 is that injuries and conditions associated with injuries, such as bone and joint issues and orthopedic disorders, are consistently among the most common conditions and are substantially more frequent than mental and psychiatric disorders. Although spacecraft contain fewer large mechanical systems than submarines, injuries are still possible, since the formation of open wounds does not rely on gravity.^17^ Indeed, hazardous incidents have already occurred on the International Space Station when high-inertia pieces of equipment were being moved in microgravity.^30^ Exposure to atmospheric contaminants also remains a concern in space; ammonia is present on the International Space Station, and one of the three space medical evacuations to date was due to smoke from a fire.^30^ Thus, space medicine should not discount the potential for injuries among astronauts, but should incorporate accident prevention and response planning as an essential part of mission protocols.^30^

Thomas and Jan’s studies also demonstrate the high prevalence of respiratory conditions among submariners, particularly upper respiratory infections,^17,28^, which are facilitated by the continual recirculation of air and the close living quarters.^29^ Similarly, Wilken notes that the number of reported sick days likely underestimates the true incidence of infectious diseases and that up to 70-90% of sailors may ultimately experience an upper respiratory infection over the course of a patrol.^29^ However, many of these respiratory infections are presumably minor, given the low use of decongestants.^18^ Furthermore, infectious diseases are not limited only to respiratory conditions; Sortor also attests to the incidence of gastrointestinal conditions, noting that foodborne norovirus infections may afflict submariners three to five times every year.^31^

Upper respiratory infections generally peak at the beginning of submarine voyages and diminish in prevalence over time, likely due to the gradual development of immunity to the strains of microbes present in the closed environment of the submarine.^15,18,32^ Hence, Thomas believes that astronauts’ mandated seven-day quarantine before spaceflight will result in a lower incidence of respiratory diseases than among submariners, who are not required to quarantine.^17^ However, *Mycoplasma pneumoniae* infections may not arise until 26 days into the voyage due to their lengthy latency period,^33^ and, in one submarine mission, an outbreak of a latent or incubating virus was observed during the third week of the voyage.^32^ Thus, submarine medicine suggests that astronauts’ quarantine period may not be long enough to resolve all of the possible issues with respiratory diseases in space. Indeed, many varieties of latent viruses can reactivate spontaneously, and such reactivations have already been noted among astronauts, including for varicella-zoster virus, herpes simplex virus, cytomegalovirus, and Epstein-Barr virus.^34,35^ In summary, these findings highlight the need for extended health monitoring for infectious diseases beyond the quarantine period, particularly during the first month of a mission.

### Mission-disruptive medical events

Due to the limited medical capabilities aboard submarines and spacecraft, severe medical conditions will undoubtedly occur that will require care on the surface of the Earth for proper treatment. In military submarines, the occurrence of such conditions is a national security issue, since surfacing the vessel to evacuate a sailor will reveal the submarine’s coordinates, jeopardizing covert military operations.^14^ Yet, it is at least technically possible to evacuate ailing submariners within a reasonable timeframe. However, in long-duration space missions, it may not be technically possible to evacuate astronauts in time for appropriate medical care, particularly if the space capsule is six months away from Earth on a trip to Mars.^4^ Thus, medical evacuations may pose greater risks in space than underwater, although they present significant challenges in both environments.

Various studies have examined the probability of emergency medical evacuations of submariners. Burr examined hospitalizations among the submarine corps; however, this information is of limited value in predicting evacuations, since the hospitalizations could have occurred either before or after the mission and were not necessarily coupled to underwater evacuations.^36^ Tansey, by contrast, provided actual data on evacuations, stratified by cause.^15^ Exactly 50% of the evacuations were ascribed to respiratory conditions (19.23%), gastrointestinal disorders (15.38%), or general surgery (15.38%), and the overall rate of evacuations was 1 per 169,615 person-days (obtained by dividing the 4,410,000 person-days by the 26 reported evacuations).

Wilken reported 14 evacuations during 360 U.S. Polaris patrols and provided vignettes of each evacuation.^29^ The most common cause was appendicitis, totaling 4 evacuations.^29^ Two instances of stroke and tuberculosis triggered evacuation, while each of the other causes was observed only once.^29^ The overall estimated rate of evacuation was 1 per 214,286 person-days (obtained by dividing the 3,000,000 person-days by the 14 evacuations).

In the 100 U.K. Polaris patrols Glover examined, there were 106 medical emergencies for which the standard of care on Earth might entail hospitalization.^14^ Nevertheless, only 1 of these incidents (a case of appendicitis) led to evacuation, and only 12 of the patients required hospital admission upon the conclusion of the voyage.^14^ Thus, the actual incidence of evacuation and hospitalization is significantly lower than the incidence of medical conditions that might otherwise require such treatment, perhaps due to the robustness of non-surgical medical care in submarines and the reluctance of crew members to surface the vessel during classified missions.

Sortor reports that the principal causes of submarine evacuations are appendicitis, closed-head trauma, abdominal pain, kidney stones, mental health emergencies, and dental issues.^31^ Sack reports that the rate of evacuation ranges from 1.9 to 2.3 per 1000 person-months,^37^, and Walton reports that there have been three evacuations thus far in space missions: a septic urinary tract infection, a cardiac dysrhythmia, and smoke inhalation.^30^ There have also been three events that nearly led to an evacuation in space: a case of nephrolithiasis, a case of dental abscess, and a case of chemical exposure.^30^ Thus, the probability of medical evacuation in space remains substantial, and, as space missions become longer and travel further from Earth, risk management for the most common causes of evacuation in the analog environment of submarines will become increasingly important. Collectively, these findings highlight a critical lesson for space missions: the importance of equipping crews with the medical tools, training, and decision-support systems necessary to manage emergencies when evacuation is not a viable option.

However, neither evacuation nor autonomous medical care may be possible in all instances, and thus the death of a crewmember cannot be completely excluded. Indeed, Wilken reported four deaths over the course of his study (1 death per 750,000 person-days).^29^ One fatality resulted from an explosion, another from suicide, a third from myocarditis, and a fourth from a brain tumor.^29^ Given this small number of deaths, it is not possible to directly compare Wilken’s distribution of actual crew deaths in submarines to Walton’s predicted probability distribution of the various causes of mortality in space.^30^ However, it is important to note that each of Wilken’s deaths was largely unpredictable: the explosion was an accident, the sailor had not previously demonstrated any suicidal inclinations, and the other two victims were in good health prior to their sudden death.^29^ Therefore, even though Wilken’s mortality rate is very small, the risk of even a single unpredictable death highlights the need for robust contingency protocols in both submarines and spacecraft, since the decomposition of the remains could induce serious health hazards for the crew.^38,39^ In this regard, NASA’s recent success in creating a durable, specialized Human Remains Containment Unit can provide a valuable asset for long-duration spaceflight, and could potentially be extrapolated to the submarine context as well.^38,39^

### Minor health problems

While Table 1 only reports diagnosed medical conditions that generate sick days, Horn reports a high prevalence of minor medical conditions that do not involve care-seeking.^18^ Indeed, in the first 52 days of a submarine voyage, he notes that 82% of sailors experience some medical incident, with nasal congestion, sleep disturbances, and back pain being the most prevalent conditions, although they rarely result in medical attention.^18^ Wilken paints a similar story: over 95% of dental and psychiatric problems did not lead to sick days, a trend that may indicate a substantial burden of self-managed or relatively minor dental and psychiatric illnesses among the crew.^29^ Furthermore, since Wilken’s study also reported a high consumption of aspirin, there may be a similarly high incidence of frequent, unreported painful conditions such as headaches.^29^ Glover also explicitly omitted minor abrasions, burns, and lacerations from his results.^14^ Thus, the true distribution of medical conditions aboard submarines may substantially differ from the distribution exhibited in Table 1, which solely presents those conditions that warranted sick days or medical diagnosis. For space missions, this consideration highlights the importance of monitoring not only formal diagnoses but also self-medicated issues which, while not mission-ending, may cumulatively impair performance and well-being.

Dermatology, vision, and oral health are three examples of such relatively minor health considerations which may require medical intervention but which can also be tackled to a significant extent through appropriate preventive care. For example, the submarine environment presents unique dermatological challenges, with a sharp increase in skin conditions, particularly cellulitis and urticaria, during the course of submarine voyages.^29^ One hypothesis for this phenomenon is the lack of ultraviolet radiation from the sun^29^ and the major decline in personal hygiene, such as showering, during patrols.^18^ Furthermore, the healing of lacerations may be negatively affected by the low levels of oxygen in the submarine atmosphere.^18,31^ Similar dermatological issues with skin rashes and slow wound healing have also been observed in space,^40,41^ suggesting a need for optimized hygiene practices and perhaps even solar-mimicking lighting to mitigate dermatological issues on long-duration missions.

Regarding vision, the enclosed environment of submarines requires sailors to constantly focus their eyes at very close distances.^42^ Some researchers have speculated that this induces myopia among submariners; a longitudinal study verified that myopia was indeed slightly greater among this population, but noted that the effect was very minor and that the precise mechanism remains unclear.^42^ While myopia has also been observed in space, optical health is further complicated by microgravity, which can harm astronauts’ vision through a syndrome termed “space flight-associated neuro-ocular syndrome” (SANS).^43–45^

Dental care is also a concern, particularly because the proportion of sailors who brush their teeth fewer than seven times per week increased from 2.5% on land to over 20% in submarines.^18^ While few dental problems aboard submarines induce sick days, Wilken reports that six conditions make up 86% of observed dental issues: (in rank order) toothaches, caries, pericoronitis/periodontitis, gingivitis, periapical abscess, and stomatitis.^29^ Fillings and extractions have been successfully carried out while underwater, although the operating conditions are not ideal.^14,29^ These dental challenges are even more severe for astronauts since microgravity also exerts negative impacts on oral health.^46^ Therefore, both submarine and space missions would benefit from enhanced attention to dermatologic, vision, and dental care to prevent these complications from arising far from the surface of the Earth and hindering mission effectiveness.

## Medical care capabilities

The preceding section has demonstrated the wide array of medical conditions that can afflict submariners and astronauts, but such data is of little use without robust medical care capabilities to treat and prevent these illnesses. Hence, this section explores this topic, beginning with a discussion of surgery in the underwater and space environments, exploring the treatment of appendicitis as a case study of the challenges with surgical care, and concluding with an examination of pharmaceutical usage in both submarines and spacecraft.

### Surgery

The naval submarine medical corps lacks the capability to perform intensive surgery. All medical care aboard the vessel is provided by a single Independent Duty Corpsman (IDC), a non-physician sailor trained for one year in basic medical care.^14,18,28^ The IDC has the authority to prescribe medicines under standing medical orders but possesses minimal surgical training, limiting their ability to perform safe surgical procedures or administer anesthesia.^14,18,28,47^ There are also challenges in procuring adequate space for both the surgery and the subsequent convalescence, and in acquiring the blood needed for transfusions, since crewmembers of the appropriate blood type must donate to the bleeding sailor.^14^

Two studies have assessed the epidemiology of surgical care in submarines, with Glover scrutinizing 100 UK patrols and Wilken evaluating 360 US patrols.^14,29^ In Column 1 of Table 2, we ranked, by order of incidence, the medical conditions presented by Glover for which surgery would typically be required and calculated the relative percentage of each.^14^ In Column 2, we conducted a parallel analysis with Wilken’s data, and, in Column 3, we calculated the relative percentages of the various surgeries performed during Wilken’s study.^29^ Column 3 reveals that only minor operations are performed aboard submarines, mainly drainage and excision procedures. However, Columns 1 and 2 demonstrate that medical crises requiring intensive surgery, such as appendicitis, fractures, burns, trauma, and severe lacerations, do occur aboard submarines, and induce sick days. However, if crewmembers requiring major surgery were evacuated, the vessel’s location would be exposed, compromising military stealth.^14,37^ Hence, IDCs are trained on the non-surgical management of surgical conditions, such as antibiotic treatment for appendicitis.^31,48^ Such treatment is frequently successful; indeed, the incidence rates we previously calculated for evacuations (1 per 169,615 person-days in Tansey’s study and 1 per 214,286 person-days in Wilken’s study) are substantially less than the rates for surgical conditions (1 per 7944 person-days in Glover’s study and 1 per 7634 person-days in Wilken’s study).^14,15,29^

**Table 2:** Surgical conditions and surgical procedures aboard submarines

Glover: Surgical Conditions (ranked by %)	Wilken: Surgical Conditions (ranked by %)	Wilken: Underwater Surgeries (ranked by %)
Appendicitis (18.87%)	Appendicitis (31%)	Drainage of Abscess (38.78%)
Abscess Formation (16.98%)	Pilonidal Cyst (19%)	Drainage of Pilonidal Cyst (17.86%)
Fractures (10.38%)	Burns (11%)	Excision of Sebaceous Cysts (10.2%)
Low Back Pain (10.38%)	Severe Laceration (9%)	Drainage of Paronychiae (8.67%)
Minor Soft Tissue Trauma (8.49%)	Hemorrhoids (5%)	Excision of Ingrown Toenail (7.14%)
Peptic Ulceration (4.72%)	Thrombophlebitis (4%)	Excision of Thrombosed External Hemorrhoids (4.59%)
Prostatitis (4.72%)	Perianal Abscess (3%)	Removal of Wart (4.59%)
Renal Colic (3.77%)	Ingrown Toenail (3%)	Removal of Nevi (2.55%)
Jaundice (2.83%)	Inguinal Hernia (2%)	Excision of Fingernail (1.53%)
Pancreatitis (1.89%)	Avulsion of Finger Tip (2%)	Drainage of Perianal Abscess (1.02%)
Inflammatory Bowel Disease (1.89%)	Infected Laceration (2%)	Incision and Drainage of Felon (0.51%)
Biliary Colic (1.89%)	Puncture Wound (2%)	Split Thickness Skin Graft (0.51%)
Cervical Spondylosis (1.89%)	Gangrene of the Toe (1%)	Excision of Clavus (0.51%)
Pilonidal Sinus (1.89%)	Hiatus Hernia (1%)	Incision and Drainage of Omphalitis (0.51%)
Cellulitis (1.89%)	Paronychia (1%)	Exploration of Inguinal Canal with Drainage of Scrotal Abscess (0.51%)
Urinary Tract Infection (1.89%)	Carbuncle (1%)	Amputation of Distal Phalanx of Toe (0.51%)
Epididymo-orchitis (1.89%)	Infected Sebaceous Cyst (1%)	-
Intestinal Obstruction (0.94%)	Abscess of Foot (1%)	-
Sialolithiasis (0.94%)	Hematoma of Thigh (1%)	-
Peritonsillar Abscess/Quinsy (0.94%)	-	-
Pneumothorax (0.94%)	-	-

The surgeries that can be performed aboard submarines are relatively minor, including drainage and excision. Nevertheless, a diversity of medical conditions that may require major surgery are present, demonstrating the necessity for robust non-surgical treatment options to minimize the risk of medical evacuations. Columns 1–2 list medical conditions; Column 3 lists procedures actually performed.

Data Sources: Glover^14^ (Table 1 – “Surgical Problems During 100 Polaris Submarine Patrols”); Wilken^29^ (Tables I (“Surgical Cases Involving Sick Days on Patrol”) and II (“Surgical Procedures Performed at Sea”)).

Yet, while non-surgical management is often adequate, it is not universally successful, and submarine mission planners must still recognize the need for surgery or medical evacuations in the event of failure. Indeed, while publicly available data is scarce, Sack reported that U.S. submarines in the Atlantic Ocean engaged in medical evacuations in 1996 at a rate of 2.0 per 1000 person-months,^37^ and Sortor stated that non-surgical management of appendicitis fails approximately 15% of the time among submariners.^31^ These results are corroborated by a recent meta-analysis in the lay population, which determined a similar recurrence rate of 18%.^49^ Sack also indicated that 63% of submarine medical evacuations could have been addressed on surface ships equipped with surgical capacities.^37^ While these studies may differ in scope, patient population, and quantitative metric, they all converge on the conclusion that non-operative management, while useful as an initial strategy, is not always sufficient.

Since 1964, when submarine physician Bruce Rice initially proposed his non-surgical protocol for managing appendicitis, there has been significant research on surgical care techniques in space, where evacuation is similarly difficult and where surgically trained crew members are also scarce.^4,19,48,50,51^ We propose that these advances can be translated into the domain of submarine medicine, facilitating surgical care below water and thus reducing the necessity for mission-endangering evacuations. For instance, NASA’s 2002-2003 Long Duration Mission Surgical Working Group determined that, after six months of focused training on a handful of important surgical procedures, any non-surgical physician could achieve the same competency in these select procedures as a surgical resident.^50^ Thus, the Navy might be able to avoid the financial expense of an accredited surgeon on each mission, while still providing quality surgical care underwater, including appendectomies, exploratory laparotomies, and cholecystectomies.^50^ Indeed, pending further training protocols and outcome validation, navies might even wish to train the IDC on surgical tasks, given that non-physician NASA astronauts, including a veterinarian and an animal physiologist, successfully conducted surgeries on rats during the STS-90 mission.^50,51^ This experiment suggests that success in performing basic surgeries within extreme environments is not limited solely to licensed physicians, but might encompass individuals with expertise in animal surgery and dissections as well.^50,51^ Jan provides further evidence in favor of a non-physician’s ability to perform the tasks of a physician, finding that IDCs correctly prescribe medications the vast majority (84%) of the time.^28^ Thus, the implications of these studies extend beyond surgery to encompass other forms of medical care as well. Indeed, based on these studies, it might be feasible to medically train a broader range of submarine personnel than just the single IDC, facilitating medical care aboard submarines even if the IDC became incapacitated during a voyage.^31,50^

Technological advances in space surgery can inform the domain of submarine surgery as well. For example, in their design for the canceled Space Station Freedom project, NASA included a robust Health Maintenance Facility equipped with the necessary technology to execute basic surgical procedures, including ventilators, an operating table, x-ray machines, and IV capabilities^50^, providing a ready-made analog for a submarine surgical suite. Furthermore, NASA has developed intravenous regional and local anesthetic capabilities that are safer and more feasible for use by non-anesthesiologists than intubated general anesthesia.^47,50^ NASA has also recognized the merits of laparoscopic surgeries within space environments, developed special surgical enclosures to preserve sterility, created a miniature ultrasound unit to improve diagnostic capabilities, and explored the use of humanoid robots to conduct surgery and 3D printing technologies to produce on-demand surgical tools.^52^ While weightlessness leads to additional challenges not present in submarines, such as blood pooling and the need to restrain the surgeon in microgravity,^50^ these developments underscore how investments in surgical training and equipment could improve contingency care in both submarine and space environments.

### Appendicitis as a case study of surgical capabilities

Since appendectomies are not available during submersion due to submarines’ limited surgical capabilities, Rice asserts that “there is a generally acknowledged fear among Navy men of appendicitis,” perhaps fueled by lore of a rogue appendectomy conducted on a submarine with kitchen spoons and mechanics’ pliers, and during which the anesthetic stopped working after one hour.^48^ While appendicitis has not yet been experienced by astronauts during space missions, it is of equal probability.^19^ Indeed, Walton reports that two members of the astronaut corps have already experienced appendicitis while still on Earth.^30^ And, in the Salyut 6 mission, a cosmonaut dreamed that he had appendicitis, showcasing the underlying anxiety of this ailment amongst space travelers.^13^

The non-surgical management of appendicitis via antibiotic therapy is frequently successful in submariners, with an efficacy of up to 85%.^31^ Nevertheless, appendectomies are still “the gold standard for treatment,” since non-surgical management can fail in approximately 30% of the cases within one year, and as often as 40% of the cases after five years.^53^ As space missions increase in duration, these recurrence rates may become quite troubling. In light of these studies, it would be prudent for submarines and spacecraft to increase their capabilities for the surgical treatment of appendicitis, whether by appendectomy or by percutaneous drainage, a technique that has been shown to be practical in zero-gravity conditions.^53^

Prophylactic appendectomies have also been proposed to eliminate the risk of appendicitis in extreme environments.^4,19,31^ These operations may be particularly beneficial among those under 35 years old, who have an elevated risk of appendicitis.^4^ They might also be advisable for the growing number of female submariners and astronauts, who may experience other abdominal symptoms that could lead to a false diagnosis of appendicitis.^4^ Nevertheless, several studies suggest that appendectomies may substantially alter the microbiome^54^ and heighten the risk for Crohn’s Disease^55,56^ and colorectal cancer.^55,57,58^ Furthermore, mandating astronauts and submariners to undergo these surgical operations raises ethical concerns.^19^ Therefore, additional research into the medical consequences of these surgeries and further contemplation of their bioethical implications would be desirable.

### Pharmaceuticals

Two studies have been conducted on the use of pharmaceuticals during submarine voyages: one (Jan) on prescription drugs and the other (Horn) on self-administered over-the-counter (OTC) drugs.^18,28^ Jan found that 77.3% of prescriptions were comprised of analgesics (mostly NSAIDs and acetaminophen), antibiotics, respiratory medicines (mostly decongestants), and skin and mucous membrane medicines (mostly anti-infectives and mouth and throat medicines).^28^ Horn similarly attests to the large numbers of OTC NSAIDs and acetaminophen, accounting for approximately one-third of all OTC drugs.^18^ Multivitamins were the most frequently taken OTC on days 1–52 and the second most common on days 53–101.^18^ Antacids and health supplements were also common.^18^ Furthermore, unlike in Jan’s prescription study, decongestants were the least common OTC at all times during the patrol, and there were no OTC antibiotics available.^18^ These patterns of pharmaceutical usage not only reflect similar burdens of disease but also point toward a high reliance on self-medication, which can inform planning for future submarine and spacecraft missions.

Wotring conducted a similar investigation of drug use in spacecraft,^59^ revealing a high prevalence of sleep aids and treatments for SAS (“space adaptation syndrome”: motion sickness).^59^ Nevertheless, despite the frequency of sleep disruptions within submariners and the possibility of motion sickness at sea, each of these drug classes is paradoxically absent in both Jan and Horn’s studies, with the exception of a single dose of an anti-vertigo drug.^18,28,59^ Therefore, the Navy might wish to consider providing sleep aids to submariners, drawing on practices in space medicine; however, Walton cautions that these drugs may produce severe side effects.^30^ Another important difference is the limited stability of drugs in space due to cosmic radiation, a concern not present in the submarine environment but which highlights the need for improved drug storage protocols in long-duration spaceflight.^60^

## Lifestyle determinants of health

The unusual lifestyle aboard submarines and spacecraft may affect multiple determinants of health. For instance, nutrition is challenged by the need to carry an adequate supply of food for the duration of the mission, exercise is hindered by the confines of the vessel, and sleep is inhibited by the unique demands of the watch cycle. Changes to the microbiome during confinement also warrant careful consideration, the accumulation of carbon dioxide in the recirculated atmosphere poses a serious concern, and the intense isolation can significantly affect the mental health of the crew. This section considers these numerous lifestyle factors and contemplates ways to reduce their negative impact on health.

### Nutrition, exercise, and weight

Submariners have difficulties maintaining a balanced diet, since the shelf life of fruits, vegetables, and dairy is shorter than typical submarine patrols.^61^ The diet they consume is very high in calories (3400–3600) and may lead to heightened serum cholesterol.^15,16^ Their risk of cardiovascular disease is further increased by disruptive sleep cycles and deficiencies in Vitamin D due to a lack of sunlight.^15,62^ Indeed, studies have noted reductions in Vitamin D of between 15 and 47% over the course of submarine voyages, although the usefulness of supplementation may vary based on the submariners’ body mass and on their baseline concentrations of Vitamin D.^63^ Nevertheless, these low levels of Vitamin D have not been shown to induce detrimental bone loss over a three-month mission,^63^ and, despite the limited opportunities for exercise in the cramped vessel, submariners do take advantage of the workout equipment, which can facilitate modest weight loss.^18^ Hence, despite the caloric diet, Horn did not report a significant increase in weight among submariners,^18^ although other studies report that many submariners are obese.^62^

While many of these nutritional and metabolic stressors are shared across both environments, spaceflight presents additional risks. Similar to submariners, astronauts’ Vitamin D levels also diminish during the mission, even while taking supplements,^8^ and their cholesterol levels may increase by 10%.^64^ However, microgravity’s impact on weight loss is unique to space medicine, inducing the atrophy of muscles and the breakdown of bones, which exercise can help to counteract.^65^ Also unique is the possible impact of space radiation and microgravity in promoting cardiovascular diseases.^66,67^ Therefore, space scientists should build on these lessons from submarine health management to tackle these unique challenges in the extraterrestrial realm.

### Sleep

The sleep of submariners is impaired for two primary reasons: the absence of a natural day-night cycle underwater and the irregularity of the watch cycle, which makes it difficult to adjust to a new rhythm of sleep and wakefulness.^68^ Indeed, sleep disturbances affected approximately one-third of submariners during Horn’s study,^18^ and, while submariners’ cortisol levels adapt to the timing of the watch cycles, their melatonin levels do not, limiting full acclimatization to the underwater lifestyle.^69^ The resultant sleep deprivation and fatigue may reduce sailors’ alertness, hindering their proficiency in responding to emergencies and in the daily task of operating the machinery aboard the complex vessel.^15,68,70^ Furthermore, sleep deprivation may impair their mood, with one study demonstrating greater anger and fatigue and reduced self-esteem among submariners after a mission compared to before.^71^ Similar issues have been noted in the context of space as well, and future missions to Mars must cope with unusual day-night cycles of 24.65 hours.^5–7^ Therefore, in both submarines and spacecraft, it is imperative for mission planners to take active steps to promote sleep, such as by introducing more regular watch cycles that mimic natural sleep patterns^68^ or by experimenting with melatonin supplementation.^69^ Indeed, empirical research has shown that the U.S. Navy’s recent modification of its 18-hour watch cycles to more natural 24-hour watch cycles has led to tangible benefits for submariners’ sleep quality, providing a potential blueprint for similar strategies in future long-duration spaceflight.^71^

### Microbiome

Spaceflight is known to impact astronauts’ microbiome, with studies demonstrating increases in opportunistic gut bacteria,^72,73^ decreases in probiotic gut bacteria,^72,73^ heightened levels of potentially pathogenic organisms in the nasal flora,^72,74^ and an increase in antibiotic resistance and virulence among the species comprising the microbiome.^72^ Far less microbiome research has been conducted among submariners, with the most recent study determining that their microbiomes are generally stable throughout a voyage, with fluctuations correlated to concurrent fluctuations in mood and diet.^75^ Most studies in submarines have focused on the microbial ecology of the vessel itself, such as the degradation of surfaces by fungal colonizers and the efficacy of ventilation systems in removing fungal spores from the atmosphere.^76–79^ However, greater research on submariners’ microbiomes is warranted, especially because the microbiota-gut-brain axis might impact the preexisting mental health issues among submariners^80^ and because the studies in space may not be directly comparable to microbial conditions aboard submarines, given the influence of microgravity in promoting the pathogenicity of microbial strains.^81^

### Carbon dioxide

Since humans continuously exhale carbon dioxide during respiration, the accumulation of this gas within enclosed vessels like spacecraft and submarines is a major concern.^15,82,83^ While the urgency of this issue was reduced after the U.S. Navy introduced improved air purifying systems in the mid-1960s,^15^ it remains a significant concern if these systems are suppressed during an emergency, when carbon dioxide levels can rise to as high as 1.8%^82^ (the ordinary level is 0.5%).^36^ Furthermore, to minimize the amount of electricity used by CO2-scrubbing equipment, the ordinary level of carbon dioxide aboard the International Space Station is purposely elevated even in non-emergency situations.^84^ High levels of carbon dioxide in a submarine or spacecraft have been shown to cause multiple adverse effects, leading to headaches,^82^ abnormally high secretions of stomach acid,^83^ and impaired decision-making and cognitive performance, a crucial concern in mission-critical environments.^85^ Therefore, in both submarine and space missions, these risks emphasize the need for renewed efforts to develop robust, energy-efficient carbon dioxide monitoring and control systems.

### Mental health

Isolation from the outside world and from one’s family and friends can lead submariners to experience motivational and emotional difficulties.^15,29,36^ Indeed, Tansey found a higher incidence of mental health conditions on submarines compared to surface ships.^15,36^ However, Burr found that the rate of mental health hospitalizations was lower in submarines than in surface ships, perhaps due to the extensive psychiatric screening required to become a submariner,^36,86^ or due to the higher evacuation threshold in submarines. To resolve this incongruity, Burr asserted that differences in Tansey’s study design between submarines and surface ships may have confounded his results.^36^ A majority of Burr’s mental health hospitalizations were caused by one of four conditions: (in rank order) alcohol abuse, personality disorders, neuroses, and drug abuse.^36^ Depression is common among sailors who are particularly devoted to their families, while anxiety often leads to headaches or insomnia, and is generally caused by worries about life after the voyage, and sometimes by self-doubt.^87^

Mental conditions during submarine missions follow a distinctive temporal trajectory that Rohrer categorized into a well-known three-part framework: “anxiety” at the beginning, “depression and boredom” in the middle, and “anticipation and jitteriness” at the end.^12,13^ Depression peaks at the commencement of the patrol,^61^ and stress is also high at the beginning of the mission, likely due to the recent separation from family and the intense work of preparing the submarine for the voyage.^87^ However, once the patrol has launched, the work diminishes and is replaced by monotony; sailors begin “counting the days.”^87^ At the conclusion of the voyage, the submariners experience “channel fever”: they are excited to return to the surface, leading to heightened energy and lapses in attention.^87^ While Rohrer's framework has proven influential in submarine psychiatry, its applicability to space missions has been questioned. For instance, studies have found no consistent evidence of a “third quarter phenomenon” among astronauts,^88^ and a longitudinal study on the International Space Station failed to detect Rohrer’s three phases,^89^ suggesting that the specific trajectories of mood patterns in isolated environments may diverge based on mission structure and environmental stressors.

Psychiatric screening can reduce the incidence of mental illness among submariners;^36,86,90^ however, many mental conditions, especially neuroses, are not immediately evident and may take several years to intensify to a level that causes disqualification.^86^ Therefore, submarine and space medicine should not rely solely on psychiatric screening, but should recognize the critical responsibility of the IDC in evaluating the psychiatric state of the crew members throughout the voyage and providing psychiatric therapies en route.^29,86^

Some of these mental health risks may be lessened in space. Indeed, as set forth above, astronauts are more akin to submarine officers than to enlisted sailors,^17^ and officers are less emotional and engage in “acting-out behavior” less frequently than their enlisted counterparts.^90^ Indeed, in Thomas’ study, no submarine officers experienced non-psychotic mental disorders, compared to 101 of the enlisted sailors.^17^ Furthermore, astronauts are performing a highly specialized role for which they were specifically trained, a factor that has been shown to positively impact mental health in submariners.^86^ However, despite these countervailing considerations, mental illness will certainly remain a serious problem in space, especially during long-distance spaceflight, such as to Mars.^9^ Indeed, mental illness is more common in ballistic missile submarines (SSBNs) than in attack submarines (SSNs), likely because of the greater potential for boredom: the submarine is underwater for longer and engages in only one task, monitoring for orders to launch its nuclear warheads.^14,86,87^ It would seem that a long-duration space mission would be similar to an SSBN voyage since the multi-month transit time is filled with little to engage the astronauts. Therefore, space agencies may wish to consider implementing the activities, recreational and otherwise, used for the mental engagement of SSBN sailors: continuous training and education, movies, and libraries.^14,87^

## Conclusion

As astronauts spend progressively more time away from Earth’s surface, the likelihood of experiencing rare medical events will inevitably increase, necessitating a greater reliance on robust, autonomous medicine.^4^ Submarine operations provide a valuable analogue to this challenge, particularly among naval officers whose demographics align closely with astronauts.^17^ Although many studies recount the incidence of diagnosed medical conditions in submarines, minor ailments likely represent the majority of medical needs aboard both vessels.^18^ Improved surgical care is one of the most impactful ways to strengthen the quality of care in both submarines and spacecraft, and, as space missions grow longer, in situ management of surgical conditions will become more and more necessary.^4^ The incidence of mental health conditions will likely also increase, given the boredom of long-distance travel.^9,14,86,87^ Improved partnerships between the submarine and space communities would have significant value in tackling this new milieu, uniting the depths and the heavens toward a joint resolution of shared problems in acute medical care. To maximize the translational value of this analogy, we suggest that future research should prioritize (1) developing cross-training programs for non-physician crew in surgical and psychiatric care, (2) investigating pharmaceutical stability in deep space conditions, and (3) quantifying minor, self-managed health issues that may cumulatively degrade mission performance. These efforts will operationalize insights from submarine medicine, helping to build more resilient and autonomous acute medical care systems for space exploration.

## Data Availability

This article is a review of previously published data. All data analyzed in this study are available within the cited publications, which are listed in the References section. No new datasets were generated or analyzed during the current study.

## References

[1] National Aeronautics and Space Administration. Artemis II. (2025).

[2] National Aeronautics and Space Administration. Moon to Mars Objectives. (2022).

[3] Krittanawong, C. et al. Human health during space travel: state-of-the-art review. *Cells* **12**, 40 (2022). 36611835 10.3390/cells12010040 PMC9818606

[4] Campbell, M. R., Johnston, S. L. 3rd, Marshburn, T., Kane, J. & Lugg, D. Nonoperative treatment of suspected appendicitis in remote medical care environments: implications for future spaceflight medical care. *J. Am. Coll. Surg.* **198**, 822–830 (2004). 15110816 10.1016/j.jamcollsurg.2004.01.009

[5] Wu, B. et al. On-orbit sleep problems of astronauts and countermeasures. *Mil. Med. Res.* **5**, 1–12 (2018). 29843821 10.1186/s40779-018-0165-6 PMC5975626

[6] Colletti, L. M. Sleep/wake cycles of personnel working a Mars day. A Thesis Presented to the Faculty of the Department of Industrial and Systems Engineering of San Jose State University (2007).

[7] Albornoz-Miranda, M., Parrao, D. & Taverne, M. Sleep Disruption, Use of Sleep-Promoting Medication, and Circadian Desynchronization in Spaceflight Crewmembers: Evidence in Low-Earth Orbit and Concerns for Future Deep-Space Exploration Missions. *Sleep. Med.: X* **6**, 100080 (2023). 37533816 10.1016/j.sleepx.2023.100080 PMC10391686

[8] Smith, S. M., Zwart, S. R., Block, G., Rice, B. L. & Davis-Street, J. E. The Nutritional Status of Astronauts Is Altered after Long-Term Space Flight Aboard the International Space Station. *J. Nutr.* **135**, 437–443 (2005). 15735075 10.1093/jn/135.3.437

[9] Alfano, C. A., Bower, J. L., Cowie, J., Lau, S. & Simpson, R. J. Long-Duration Space Exploration and Emotional Health: Recommendations for Conceptualizing and Evaluating Risk. *Acta Astronaut.* **142**, 289–299 (2018).

[10] Cherry, J., Ayton, J., Cooper, D. & Zosky, G. A systematic review of medical emergencies in Antarctica: Lessons for long duration spaceflight. *Acta Astronaut* **225**, 328–336 (2024).

[11] Todd, B. & Reagan, M. in Engineering, Construction, and Operations in Challenging Environments: Earth and Space 2004 751–758, (2004).

[12] Rohrer, J. H. Interpersonal relationships in isolated small groups. *Psychophysiol. Aspects Space Flight*, 263–271 (1961).

[13] Kanas, N. Psychological, psychiatric, and interpersonal aspects of long-duration space missions. *J. Spacecr. Rockets* **27**, 457–463 (1990). 10.2514/3.26165 11537616

[14] Glover, S. D. & Taylor, E. W. Surgical problems presenting at sea during 100 British Polaris submarine patrols. *J. R. Naval Med. Serv* **67**, 65–69 (1981). 7310747

[15] Tansey, W. A., Wilson, J. M. & Schaefer, K. E. Analysis of health data from 10 years of Polaris Submarine Patrols. *Undersea Biomed. Res.* **6**, 217 (1979). 505628

[16] Tappan, D. V., Jacey, M. J. & Heyder, E. Biochemical and hematologic profiles of 1000 submariners. *Undersea Biomed. Res. Sub. Suppl*., S191–S199 (1979) https://apps.dtic.mil/sti/html/tr/ADA076225/. 505626

[17] Thomas, T. L. et al. Health of U.S. Navy Submarine Crew During Periods of Isolation. *Aviat., space, Environ. Med.* **74**, 260–265 (2003). 12650274

[18] Horn, W. G., Thomas, T. L., Marino, K. & Hooper, T. I. Health Experience of 122 Submarine Crewmembers During a 101-Day Submergence. *Aviat., space, Environ. Med.* **74**, 858–862 (2003). 12924761

[19] Ball, C. G. et al. Prophylactic surgery prior to extended-duration space flight: Is the benefit worth the risk?. *Can. J. Surg.* **55**, 125 (2012). 22564516 10.1503/cjs.024610 PMC3310768

[20] Hellweg, C. E., Berger, T., Matthiä, D. & Baumstark-Khan, C. in *Radiation in space: relevance and risk for human missions* (Springer, 2020).

[21] Marchal, S. et al. Challenges for the human immune system after leaving Earth. *npj Microgravity* **10**, 106 (2024). 39557881 10.1038/s41526-024-00446-9 PMC11574097

[22] Hicks, J., Olson, M., Mitchell, C., Juran, C. M. & Paul, A. M. The impact of microgravity on immunological states. *Immunohorizons* **7**, 670–682 (2023). 37855736 10.4049/immunohorizons.2200063 PMC10615652

[23] Le Roy, B. et al. Is sleep affected after microgravity and hypergravity exposure? A pilot study. *J. Sleep. Res.* **34**, e14279 (2025). 38923005 10.1111/jsr.14279 PMC11911056

[24] Lyu, Q. et al. Simulated microgravity predisposes kidney to injury through promoting intrarenal artery remodeling. *FASEB J.* **39**, e70353 (2025). 39874067 10.1096/fj.202402267R

[25] Shah, J. et al. Risk of Permanent Corneal Injury in Microgravity: Spaceflight-Associated Hazards, Challenges to Vision Restoration, and Role of Biotechnology in Long-Term Planetary Missions. *Life* **15**, 602 (2025). 40283157 10.3390/life15040602 PMC12028470

[26] The Sun UK. PM Honours Navy Submarine Crew Who Broke World Record After Spending 204 Days At Sea. (2025).

[27] National Aeronautics and Space Administration. Mars Transportation Architecture Concept Review 2022 White Paper. (2022).

[28] Jan, M. H., Thomas, T. L. & Hooper, T. I. Prescription medicine use aboard US submarines during periods underway. *Undersea Hyperbaric Med.* **29**, 294 (2002). 12797671

[29] Wilken, D. D. Significant Medical Experiences Aboard Polaris Submarines: A Review of 360 Patrols During the Period 1963-1967. *U.S. Naval Submarine Medical Center* report **560** (1969).

[30] Walton, M. E. & Kerstman, E. L. Quantification of Medical Risk on the International Space Station Using the Integrated Medical Model. *Aerosp. Med Hum. Perform.* **91**, 332–342 (2020). 32493555 10.3357/AMHP.5432.2020

[31] Doarn, C. R., Pantalos, G., Strangman, G. & Broderick, T. J. Surgical Capabilities for Exploration and Colonization Space Flight - An Exploratory Symposium, National Space Biomedical Research Institute, Houston, TX, 2016-11.

[32] Watkins, H. M. S. Epidemiologic investigations in Polaris submarines. *Aerobiology*, 9–53 (1970).

[33] Sawyer, R. & Sommerville, R. G. An outbreak of Mycoplasma pneumoniae infection in a nuclear submarine. *JAMA* **195**, 958–959 (1966). 5951973

[34] Mehta, S. K. et al. Multiple latent viruses reactivate in astronauts during Space Shuttle missions. *Brain Behav. Immun.* **41** (2014). 10.1016/j.bbi.2014.05.014 24886968

[35] Mehta, S. K. et al. Reactivation of latent viruses is associated with increased plasma cytokines in astronauts. *Cytokine* **61** (2012). 10.1016/j.cyto.2012.09.019 23107825

[36] Burr, R. G. & Palinkas, L. A. Health Risks Among Submarine Personnel in the U.S. Navy, 1974-1979. *Naval Health Res. Center* (1987). 3686743

[37] Sack, D. M. U.S. Navy Submarine Medical Evacuations: 1993-1996 - An Epidemiologic Assessment (UHMS Annual Scientific Meeting Sessions, Seattle, Washington, May 21-23, 1998).

[38] National Aeronautics and Space Administration. Mortality Related to Human Spaceflight: NASA-STD-3001 Technical Brief OCHMO-TB-012 Rev A. (2023).

[39] Travis Houser. Operational Considerations for Death in Space: Hardware Considerations for Preparation, Stowage, and Potential Return of Remains. National Aeronautics and Space Administration. (2021).

[40] Braun, N., Thomas, S., Tronnier, H. & Heinrich, U. Self-Reported Skin Changes by a Selected Number of Astronauts after Long-Duration Mission on ISS as Part of the Skin B Project. *Skin Pharmacol. Physiol.* **32** (2018). 10.1159/000494689 30485842

[41] Crucian, B. et al. A case of persistent skin rash and rhinitis with immune system dysregulation onboard the International Space Station. *J. Allergy Clin. Immunology: Pract.* **4**, 759–762 (2016). 27036643 10.1016/j.jaip.2015.12.021

[42] Kinney, J. A. S. The vision of submariners and National Guardsmen: a longitudinal study. *Optom. Vis. Sci.* **57**, 469–478 (1980). 10.1097/00006324-198008000-00001 7457562

[43] Seedhouse, E. in Microgravity and Vision Impairments in Astronauts. (Springer International Publishing, 2015).

[44] Waisberg, E. et al. Addressing Empty Space Myopia to Enable Deep Space Travel with Extended Reality Auditory Biofeedback. *Int. J. Aviation Aeron. Aerospace* **11** (2024).

[45] Lee, A. G., Mader, T. H., Gibson, C. R., Brunstetter, T. J. & Tarver, W. J. Space Flight-Associated Neuro-Ocular Syndrome (SANS). *Eye* **32**, 1164–1167 (2018). 29527011 10.1038/s41433-018-0070-y PMC6043524

[46] Hasin Abdul et al. Smile beyond the stars: a narrative review exploring the challenges for dentistry in space. *Cureus* **16** (2024). 10.7759/cureus.66591 PMC11383425 39252744

[47] Komorowski, M. in Precision Medicine for Long and Safe Permanence of Humans in Space. 151–162 (Elsevier, 2025).

[48] Rice, B. H. Conservative, non-surgical management of appendicitis. *Mil. Med.* **129**, 903–920 (1964). 14209537

[49] de Almeida Leite, R. M. et al. Nonoperative vs operative management of uncomplicated acute appendicitis: a systematic review and meta-analysis. *JAMA Surg.* **157**, 828–834 (2022). 35895073 10.1001/jamasurg.2022.2937 PMC9330355

[50] Barratt, M. R., Baker, E. S. & Pool, S. L. in Principles of Clinical Medicine for Space Flight. (Springer Science & Business Media, New York, NY, 2008).

[51] Campbell, M. R., Williams, D. R., Buckey, J. C. & Kirkpatrick, A. W. Animal Surgery During Spaceflight on the Neurolab Shuttle Mission. *Aviat. Space Environ. Med.* **76**, 589–593 (2005). 15945406

[52] Rajput, S. A review of space surgery-What have we achieved, current challenges, and future prospects. *Acta Astronaut* **188**, 18–24 (2021).

[53] Barie, P. S. Non-Operative Management of Appendicitis: Evolution, not Revolution. *Surg. Infect.* **22**, 991–1003 (2021). 10.1089/sur.2021.059 34546087

[54] Sánchez-Alcoholado, L. et al. Incidental Prophylactic Appendectomy Is Associated with a Profound Microbial Dysbiosis in the Long-Term. *Microorganisms* **8**, 609 (2020). 32340272 10.3390/microorganisms8040609 PMC7232405

[55] Dilek, O. N., Uranues, S. & Latifi, R. in *Prophylactic Surgery* (Springer Nature Switzerland AG, 2021).

[56] Zhang, L. et al. Association between prior appendectomy and the risk and course of Crohn’s disease: a systematic review and meta-analysis. *Clin. Res. Hepatol. Gastroenterol.* **47**, 102090 (2023). 36746236 10.1016/j.clinre.2023.102090

[57] Liu, Z., Ma, X., Zhu, C. & Fang, J. - Risk of Colorectal Cancer After Appendectomy: A Systematic Review and Meta-Analysis. *J. Gastroenterol. Hepatol.* **38**, 350–358 (2023). 36637673 10.1111/jgh.16108

[58] Wu, S. et al. Association between Appendectomy and Subsequent Colorectal Cancer Development: An Asian Population Study. *PLoS ONE* **10**, e0118411 (2015). 25710790 10.1371/journal.pone.0118411 PMC4339380

[59] Wotring, V. E. Medication use by U.S. crewmembers on the International Space Station. *FASEB J.* **29**, 4417–4423 (2015). 26187345 10.1096/fj.14-264838

[60] Mehta, P. & Bahyani, D. Impact of Space Environment on Stability of Medicines: Challenges and Prospects. *J. Pharm. Biomed. Anal.* **136**, 111–119 (2017). 28068518 10.1016/j.jpba.2016.12.040

[61] Reynolds, R. D., David, D. J. & Schlichting, C. L. Decreased vitamin B-6 status of submariners during prolonged patrol. *Am. J. Clin. Nutr.* **47**, 463–469 (1988). 3348157 10.1093/ajcn/47.3.463

[62] Mahmood, R. et al. Navigating the Depths of Cardiovascular Effects on Submariners. *Cardiol. Rev.* (2024) https://journals.lww.com/cardiologyinreview/fulltext/9900/navigating_the_depths_of_cardiovascular_effects_on.317.aspx. 10.1097/CRD.0000000000000780 39194202

[63] Gasier, H. G. et al. The efficacy of vitamin D supplementation during a prolonged submarine patrol. *Calcif. Tissue Int.* **95**, 229–239 (2014). 25005834 10.1007/s00223-014-9886-z

[64] Smith, S., Heer, M. & Zwart, S. Nutrition and Human Space Flight: Evidence From 4-6 Month Missions to the International Space Station. *Curre. Dev. Nutr.* 863 (2021).

[65] Matsumoto, A. et al. Weight Loss in Humans in Space. *Aviat. Space Environ. Med.* **82** (2011). 10.3357/asem.2792.2011 21702312

[66] Delp, M. D., Charvat, J. M., Limoli, C. L., Globus, R. K. & Ghosh, P. Apollo lunar astronauts show higher cardiovascular disease mortality: possible deep space radiation effects on the vascular endothelium. *Sci. Rep.* **6**, 29901 (2016). 27467019 10.1038/srep29901 PMC4964660

[67] Krittanawong, C., Isath, A., Kaplin, C. & Lavie, C. J. Cardiovascular Disease in Space: A Systematic Review. *Prog. Cardiovasc. Dis.* **81**, 33–41 (2023). 37531984 10.1016/j.pcad.2023.07.009

[68] Kleitman, N. The sleep-wakefulness cycle of submarine personnel. *Hum. Fact. Undersea Warfare* 329–341 (1949).

[69] Van Puyvelde, M. et al. The submariners’ sleep study: a field investigation of sleep and circadian hormones during a 67-day submarine mission with a strict 6-h-on/6-h-off watch routine. *J. Appl. Physiol.* **132**, 1069–1079 (2022). 35142558 10.1152/japplphysiol.00130.2021

[70] Duplessis, C. A., Miller, J. C., Crepeau, L. J., Osborn, C. M. & Dyche, J. Submarine watch schedules: underway evaluation of rotating (contemporary) and compressed (alternative) schedules. *Undersea Hyperbaric Med.* **34**, 21 (2007). 17393936

[71] Chabal, S. A., Markwald, R. R. & Chinoy, E. D. Life onboard a submarine: Sleep, fatigue, and lifestyle behaviors of sailors on a circadian-aligned watchstanding schedule. *Appl. Ergon.* **119**, 104321 (2024). 38820921 10.1016/j.apergo.2024.104321

[72] Tesei, D., Jewczynko, A., Lynch, A. M. & Urbaniak, C. Understanding the complexities and changes of the astronaut microbiome for successful long-duration space missions. *Life* **12**, 495 (2022). 35454986 10.3390/life12040495 PMC9031868

[73] Liu, Z. et al. Effects of spaceflight on the composition and function of the human gut microbiota. *Gut Microbes* **11**, 807–819 (2020). 31924114 10.1080/19490976.2019.1710091 PMC7524348

[74] Nefedov, Y. G., Shilov, V. M., Konstantinova, I. V. & Zaloguyev, S. N. Microbiological and immunological aspects of extended manned space flights. *Life Sci. space Res.* **9**, 11–16 (1971). 12206177

[75] Liechty, Z. S. et al. *Meeting report of the seventh annual Tri-Service Microbiome Consortium Symposium* (BMC proceedings Ser. 18, Springer, 2024). 10.1186/s12919-024-00307-z PMC11542233 39506745

[76] Upsher, J. F., Fletcher, L. E. & Upsher, C. M. Microbiological conditions on Oberon submarines. Australian Department of Defense Defense Science & Technological Organization. (1994).

[77] Milroy, W. C. & Duffner, G. J. The Fungal Flora of the Submarine Environment During Prolonged Submergence. *United States Naval Submarine Medical Research Laboratory* Report No. **535** (1968).

[78] Doris, I., Harper, M. G. & Morris, J. E. W. The significance of fungal growth in nuclear submarines. *J. R. Nav. Med. Serv.* **58**, 123–127 (1972). 5081689

[79] Prucka, A. P., Bennett, J. W. & Cordero, R. J. mGem: Submarine Mycology - An Analog to Astromycology. *mBio* **16** (2025). 10.1128/mbio.01686-25 PMC12607643 40985658

[80] Liu, R. T. The microbiome as a novel paradigm in studying stress and mental health. *Am. Psychol.* **72**, 655 (2017). 29016169 10.1037/amp0000058 PMC5637404

[81] Simões, M. F. & Antunes, A.0 Microbial pathogenicity in space. *Pathogens* **10** (2021). 10.3390/pathogens10040450 PMC8069885 33918768

[82] Demers, G., Horn, W. G., Linda, M. & Hughes. Assessment of Headache Incidence During SURVIVEX 2004. Naval Submarine Research Laboratory. (2009).

[83] Schaefer, K. E. Effects of Increased CO_2_ Levels on Human and Animal Health. *Experientia* **38**, 1163–1168 (1982). 6814945 10.1007/BF01959726

[84] Georgescu, M. R., Meslem, A. & Nastase, I. Accumulation and spatial distribution of CO_2_ in the astronaut’s crew quarters on the International Space Station. *Build. Environ.* **185**, 107278 (2020).

[85] Du, B., Tandoc, M. C., Mack, M. L. & Siegel, J. A. Indoor CO_2_ concentrations and cognitive function: a critical review. *Indoor Air* **30**, 1067–1082 (2020). 32557862 10.1111/ina.12706

[86] Weybrew, B. B. & Noddin, E. M. Psychiatric Aspects of Adaptation to Long Submarine Missions. *Aviat., Space, Environ. Med.* **50**, 575–580 (1979). 475704

[87] Serxner, J. L. An Experience in Submarine Psychiatry. *Am. J. Psychiatry* **125**, 25–30 (1968). 5657372 10.1176/ajp.125.1.25

[88] Kanas, K., Gushin, V. & Yusupova, A. Whither The Third Quarter Phenomenon? *Aerospace Med. Hum. Perform.* **92** (2021). 10.3357/AMHP.5857.2021 34503622

[89] Kanas, N. A., Salnitskiy, V. P., Boyd, J. E., Gushin, V. I. & Marmar, C. I. Crewmember and Mission Control Personnel Interactions During International Space Station Missions. *Aviat. Space Environ. Med.* **78** (2007). 17571662

[90] Weybrew, B. B. & Noddin, E. M. The mental health of nuclear submariners in the United States Navy. *Mil. Med.* **144**, 188–191 (1979). 107489

